# DIAPH3 is a prognostic biomarker and inhibit colorectal cancer progression through maintaining EGFR degradation

**DOI:** 10.1002/cam4.4793

**Published:** 2022-05-11

**Authors:** Renli Huang, Cheng Wu, Jialing Wen, Jianyang Yu, Huidong Zhu, Jinlong Yu, Zhaowei Zou

**Affiliations:** ^1^ Department of General Surgery, Zhujiang Hospital Southern Medical University Guangzhou Guangdong China; ^2^ Department of Gastroenteric Hernia Ganzhou People's Hospital Ganzhou Jiangxi China; ^3^ Guangdong Provincial Key Laboratory of Colorectal and Pelvic Floor Disease, The Sixth Affiliated Hospital Sun Yat‐sen University Guangzhou Guangdong China; ^4^ Department of General Surgery Ruijin People's Hospital Ganzhou Jiangxi China

**Keywords:** actin cytoskeleton, colorectal cancer, DIAPH3, EGFR, prognosis

## Abstract

**Background:**

Actin cytoskeleton is connected with the processes of cell proliferation and migration in colorectal cancer (CRC). However, it is unknown how to accomplish these adjustments in CRC by actin cytoskeleton genes (ACGs) and here we investigated the role of hub prognosis‐related ACGs‐Diaphanous‐related formin 3 (DIAPH3) in CRC, as a potential, novel target.

**Methods:**

The ACGs gene set from the Kyoto Encyclopedia of Genes and Genomes (KEGG) was used to group CRC patients and select prognosis‐related ACGs by univariate and multivariate Cox regression for constructing prognostic model. Next, we tested hub prognosis‐related ACGs‐ DIAPH3 expression in CRC and clarified the role of DIAPH3 by shRNA constructs in KM12 and SW480. Activation of EGFR was analyzed by western blot and immunofluorescence.

**Results:**

The results showed that actin cytoskeleton function is a significant prognostic factor for CRC patients and related to clinicopathological characteristics such as T stage and lymph node metastasis. A prognostic model constructed by four prognosis‐related ACGs has a moderate intensity to 1‐year Survival (AUC = 0.71). And hub prognosis‐related ACGs DIAPH3 is downregulated in CRC. Knockdown of DIAPH3 could promote the proliferation and migration capacity of CRC. In addition, DIAPH3‐silenced cells increase EGFR phosphorylation by inhibiting EGFR transportation to lysosome.

**Conclusions:**

ACGs play a significant role in tumor invasion and have the potential to predict the prognosis of CRC. Prognosis‐related ACGs DIAPH3 might be a new prognostic biomarker and DIAPH3 could inhibit CRC progression through maintaining EGFR degradation.

## Graphical abstract

The regulation of the actin cytoskeleton is intimately connected with the processes of cell proliferation and migration. Our study shows that actin cytoskeleton genes (ACGs) play a significant role in tumor invasion and have the potential to predict the prognosis of colorectal cancer (CRC). Hub prognosis‐related ACGs DIAPH3 might be a new prognostic biomarker and DIAPH3 could inhibit CRC progression through maintaining EGFR degradation.

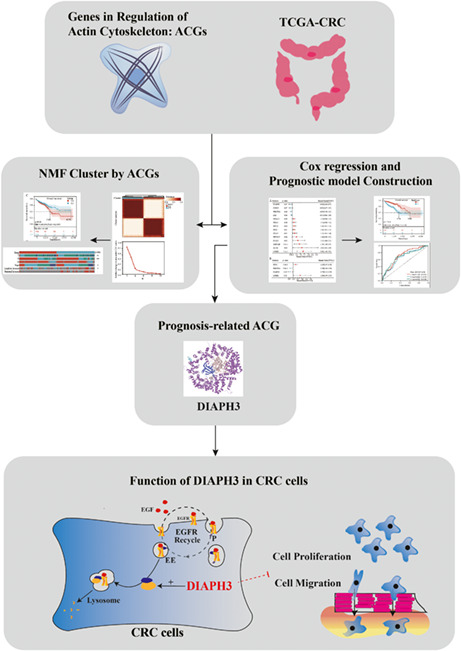

## 1 INTRODUCTION

CRC is the most prevalent malignant tumor.^1^ Tumor development and metastasis are significant determinants in the mortality rate from CRC. The regulation of the actin cytoskeleton is intimately connected with the processes of cell proliferation and migration, including the formation and adhesion of pseudopodia in the formation and migration of the cell division spindle, and serves as a neckless for tumor cells to acquire migration ability.

Numerous initiators of CRC, such as P53^2^ and APC,^3^ are involved in the remodeling process of actin cytoskeletons. Among these, APC mutations disrupt the spindle formation and orientation mechanisms during cell division, resulting in asymmetric division and uncontrolled proliferation.^3^ P53 controls the actin nucleation factor transcription process, allowing CRC cells to adapt to the mechanical stress of the tumor microenvironment and thereby promote cancer growth.^4^ Thus, it is critical to understand the role of actin cytoskeletal control in the course and prognosis of CRC and to look for important regulators for CRC‐targeted therapy. In CRC, the mechanism by which the actin cytoskeleton promotes proliferation and migration is not completely understood. We split CRC patients into two groups based on the expression of ACGs and screened for the crucial factor DIAPH3 in this research.

DIAPH3 is a key member of the formin family, which serves as the major effector of the Rho GTPase.^5^ Its core area FH1‐FH2 domain regulates microtubule polymerization and actin filament extension, which are required for cell motility.^6^ Additionally, DIAPH3 participates in cell division,^7^ cell morphological differentiation, intracellular transport, and vesicle transport.^8^, ^9^ Researches indicate that DIAPH3 deficiency has been linked to tumor invasion and metastasis in breast cancer and prostate cancer.^10^, ^11^ However, there are contradicting findings in lung and pancreatic cancer, showing that DIAPH3 can promote tumor progression.^12^, ^13^ Its mechanism for controlling the growth and migration of CRC has yet to be discovered. As a result, we started by examining the relationship between DIAPH3 and tumor invasion and metastasis, with the goal of elucidating the mechanism by which it functions in CRC.

In this study, we utilized TCGA data to demonstrate that regulation of the actin cytoskeleton is associated with tumor development and prognosis in CRC. Here, we have characterized one of these ACG‐DIAPH3, which is received relatively little attention in CRC. Surprisingly, knocking down DIAPH3 greatly increased cell proliferation, migration, and influences EGFR degradation. Thus, our findings support the possibility that DIAPH3 depletion also prevents EGFR degradation and constantly activating EGFR to enhance cell proliferation and migration in CRC. Consequently, DIAPH3 could be a potential biomarker and a promising therapeutic target in CRC. Clarifying DIAPH3 mechanism can better explore the critical role of actin cytoskeleton in tumor development and metastasis of CRC.

## 2 MATERIALS AND METHODS

### 2.1 Patients and samples

In this study, we included 617 patients identified in The Cancer Genome Atlas‐Colorectal Adenocarcinoma and Rectum Adenocarcinoma (TCGA‐CRC) (https://portal.gdc.cancer.gov/). Raw RNA‐sequencing data counts (level 3) of patients in the TCGA‐CRC cohort were downloaded from the TCGA database as recommended by guidelines, and converted into transcripts per kilobase million for analysis.

CRC specimens and adjacent normal tissues were collected from six patients who underwent CRC surgery. The included patients did not receive any preoperative antitumor therapy, including radiotherapy, chemotherapy, or targeted treatment. All fresh tissues were harvested on the ice and placed in liquid nitrogen in a 1.5 ml EP tube for transfer and eventually stored at −80°C. Another part of the specimens was immersed in formalin for immunohistochemistry.

### 2.2 Immunohistochemistry

CRC tumor tissue and adjacent normal tissue sections were deparaffinized in xylene, repaired with citrate antigen (Code: Beyotime, Shanghai, China), blocked in 1% goat serum at room temperature for 1 h, and then incubated at 4°C overnight with the DIAPH3 antibody (Code: 14342‐1‐AP, 1:100, Proteintech, Wuhan, China). Secondary antibody was applied to incubate slides, stained with the DAB Kit (Code: ZLI‐9018, ZSGB‐BIO, Beijing, China) and restrained with hematoxylin. The image was captured under a microscope for analysis. Tumor sample with a score of >4 was defined as positive, and scores between 1 and 4 were considered weak expressions for DIAPH3, while others were regarded as negative expressions.

### 2.3 Cell culture

Seven human CRC cell lines (LoVo, HCT116, HCT8, RKO, SW480, and KM12) and the normal colon epithelial cell line NCM460 were donated by Dr Jialing Wen (Gastroenterology Institute, Affiliated Sixth Hospital, Sun Yat‐sen University, Guangzhou, China). All cell lines were cultured in Roswell Park Memorial Institute (RPMI) 1640 medium (Code: C11875500BT, Gibco, USA) with 10% fetal bovine serum (Code: 10270–106, Gibco, USA), 100 μg/ml streptomycin, and 100 U/ml penicillin in a 5% CO_2_ atmosphere at 37°C. Intramimic‐01 (IMM‐01) (Code: SML1064, Sigma‐Aldrich, USA) and gefitinib (Code: HY‐50895 ‐100 mg, MCE, USA) (1 μM) were added to the cultures for research.

### 2.4 Plasmid construction and cell transfection

Constructing *E. coli* carrier with DIAPH3 RNA interference (WeiZhen, Shandong, China) and using a EZgene™ EndoFree Plasmid Miniprep Kit (Code: PD1212‐01, BIOMIGA, USA) to extract target gene plasmids. Next, lentiviral vector was constructed by mixing the target gene plasmid with pMD2G and pSPA2X vector plasmids. Lentivirus containing sh‐DIAPH3 or sh‐NC was transfected into 293 T cells. Then collect the cultured supernatant to transfect KM12 and SW480 cell lines. The RNA interference sequence listed in Supplementary Data S1.

### 2.5 RNA extraction and qRT‐PCR

After culturing those two cell lines in 10% FBS 1640 serum with 4 μg/ml puromycin for 2 weeks, total RNA from each group (sh‐NC and sh‐DIAPH3) was extracted with TRIzol® Reagent Life (Code: 15596026, Ambion, USA) to convert to cDNA (Code: FSQ‐301, TOYOBO, Japan) for qRT‐PCR (CFX96, Bio‐Rad, USA). The PCR reaction contained 1 μl of cDNA, 0.4 μl of forward primer, 0.4 μl of reverse primer, 3.2 μl of DEPC H_2_O, and 5 μl of ChamQ Universal SYBR qPCR Master Mix (Code: Q711‐02, Vazyme, Nanjing, China) with conditions set as 95°C 30 s, 1 cycle; 95°C 5 s; 60°C 30 s, 40 cycle; 95°C 15 s, 60°C 60 s, 95°C 15 s, 1 cycle. 2‐ΔΔCT was used to calculate RNA expression with an internal control of GAPDH. The primers are listed in Supplementary Data S1.

### 2.6 Western blot

Proteins were extracted from frozen colorectal tissues and cultured cells using RIPA solution (Code: P0013B, Beyotime, Shanghai) and a protease inhibitor (Code: A32961, Thermo Fisher Scientific, Shanghai, China), NaF (50 mM), PMSF (1 mM), and Na3VO4 (1 mM). All samples were lysed at 4°C after 30 min. The mixtures were then centrifuged at  12,000 ***g*** for 15 min at 4°C, and the supernatant was carefully collected. Next, we used the PierceTM BCA Protein Assay Kit (Code: 23227, Thermo Fisher Scientific, Shanghai, China) to measure the protein expression level by absorbance at 570 nm. Then, 30 μg of each protein sample was separated by 10% SDS‐PAGE and electrophoretically transferred to polyvinylidene fluoride membranes. To block nonspecific protein binding, use 5% DifcoTM Skim Milk (Code: 232100, BD, USA) dissolved in TBST overnight at 4°C. Membranes were incubated with primary antibodies overnight at 4°C. After being washed three times with TBST buffer, HRP‐conjugated IgG or IgM (working dilutions: 1:1000; Code: A200 and A21, Beyotime, China) as secondary antibodies, and incubated at room temperature for 1 hour. Antigen and antibody complexes were detected following a Bio‐Rad ChemiDoc™ Touch. Immunoblots were quantified using ImageJ (Quantity One software, Bio‐Rad, CA, USA). All primary antibodies are listed in Supplementary Data S1.

### 2.7 Cell migration assay

KM12 and SW480 cells transfected with sh‐DIAPH3 or sh‐NC were harvested after being cultured without FBS for 24 h (serum‐free medium). The cells were resuspended in serum‐free medium and diluted to 2.5 × 10^5^ cells/ml, and 200 μl of cell suspension was plated into the upper chambers of Transwell inserts (Code: 3422, Corning, NY, USA) for migration (without Matrigel). The inserts were placed in 24‐well plates containing 700 μl 1640 medium with 10% serum. After incubation for 24 h for migration, cells were fixed with 4% polyformaldehyde for 30 min and stained with 0.1% crystal violet for 30 min. Images of each Transwell chamber were captured, and cell numbers were calculated from five random fields.

### 2.8 Colony‐forming assay

KM12 and SW480 cells transfected with sh‐DIAPH3 or sh‐NC were fully suspended to make a single cell suspension, and 250 cells were plated in a 6‐well plate and cultured for 2 weeks with medium changed every 2 days. After washing with PBS, cells were fixed with 4% polyformaldehyde for 30 min and stained with 0.1% crystal violet for 30 min. Images were captured for counting under a light microscope.

### 2.9 Wound healing assays

Cells (1 × 10^5^ cells/90 μl of 10% FBS medium cell suspension) were injected to each side of the culture insert (Code: 80241, IBIDI, Germany) fixed in 6‐well plates. After cell attachment, we removed the culture insert and washed the cells with PBS, then cultured them in serum‐free medium at 37°C with 5% CO_2_. Images were captured under a light microscope at 0, 24, and 48 h and measured the wound area with the software ImageJ.

### 2.10 Cell proliferation assays

Cell counting kit‐8 assay was used to estimate cell proliferation. Cell suspension of 2 × 10^3^ cells /100 μl was added into each well of a 96‐well plate. When cells were cultured in 10% FBS medium for 6, 24, 48, 72, 96, or 120 h, 10 μl CCK‐8 solution (Code: CK04, DOJINDO, Japan) was added to each well of the 96‐well plate and incubated for 2 h in the dark at 37°C. Absorbance at 450 nm was measured in multimode microplate reader to assess cell counts.

### 2.11 Immunofluorescence

For immunofluorescence staining, seed the above cells on a coverslip of a 6‐well plate (30,000 cells per well), adhere to the wall and fix them in 4% PFA. The cells are then blocked for 1 h with 0.1% TritonX‐100 in 5% goat serum before being incubated overnight at 4°C with EGFR primary antibody, LAMP1 primary antibody, and EEA1 primary antibody (1:100). Fluorescein‐labeled and Alexa Fluor 488 and 555‐labeled secondary antibodies are used to process cells for 1 h. Finally, DAPI is used to stain and fix the coverslip, which is then looked at by a laser scanning confocal microscope. ImageJ was used to perform colocation analysis with the primary objective of comparing Pearson's correlation coefficient of red and green fluorescent dots among cells by Coloc 2.^14^

### 2.12 TMT‐based quantitative proteomics

Ten million cells from each sample transfected with sh‐DIAPH3 or sh‐NC were harvested. For sample lysis and protein extraction, SDT (4% SDS, 100 mM Tris–HCl, 1 mM DTT, pH 7.6) buffer was used. Peptide mixture (100 μg) of each sample was labeled using TMT reagent according to the manufacturer's instructions (Thermo Scientific), and LC–MS/MS analysis was performed on a Q Exactive mass spectrometer (Thermo Scientific). The MS raw data for each sample was searched using the MASCOT engine (Matrix Science, London, UK; version 2.2) embedded into Proteome Discoverer 1.4 software for identification and quantitation analysis (Supplementary Table S2).

### 2.13 Gene Ontology (GO) and Kyoto Encyclopedia of Genes and Genomes (KEGG) enrichment analysis

For the functional enrichment analysis of gene sets, the GO annotations of genes in the R software package org.hs.eg. db (Version 3.1.0) and KEGG annotations of genes in the API (https://www.kegg.jp/kegg/rest/keggapi.html) were used as the background set. R software package cluster Profiler (Version 3.14.3) was used to map genes into the background. An enrichment analysis was done to determine the result of gene set enrichment. The minimum and maximum gene sets were set at 5 and 5000, respectively. *p* value of 0.05 and FDR of 0.25 were considered statistically significant.

### 2.14 Statistical analysis and quantification

GraphPad Prism 8.0 and Statistical Program for Social Sciences 20.0 were used for statistical analysis. ANOVA and *t*‐tests were applied for analysis with continuous variables. Experiments were repeated in triplicate. *p* < 0.05 indicated statistically significant differences.

## 3 RESULTS

### 3.1 Definition of CRC subgroup by gene set from Regulation of actin cytoskeleton

**FIGURE 1:**
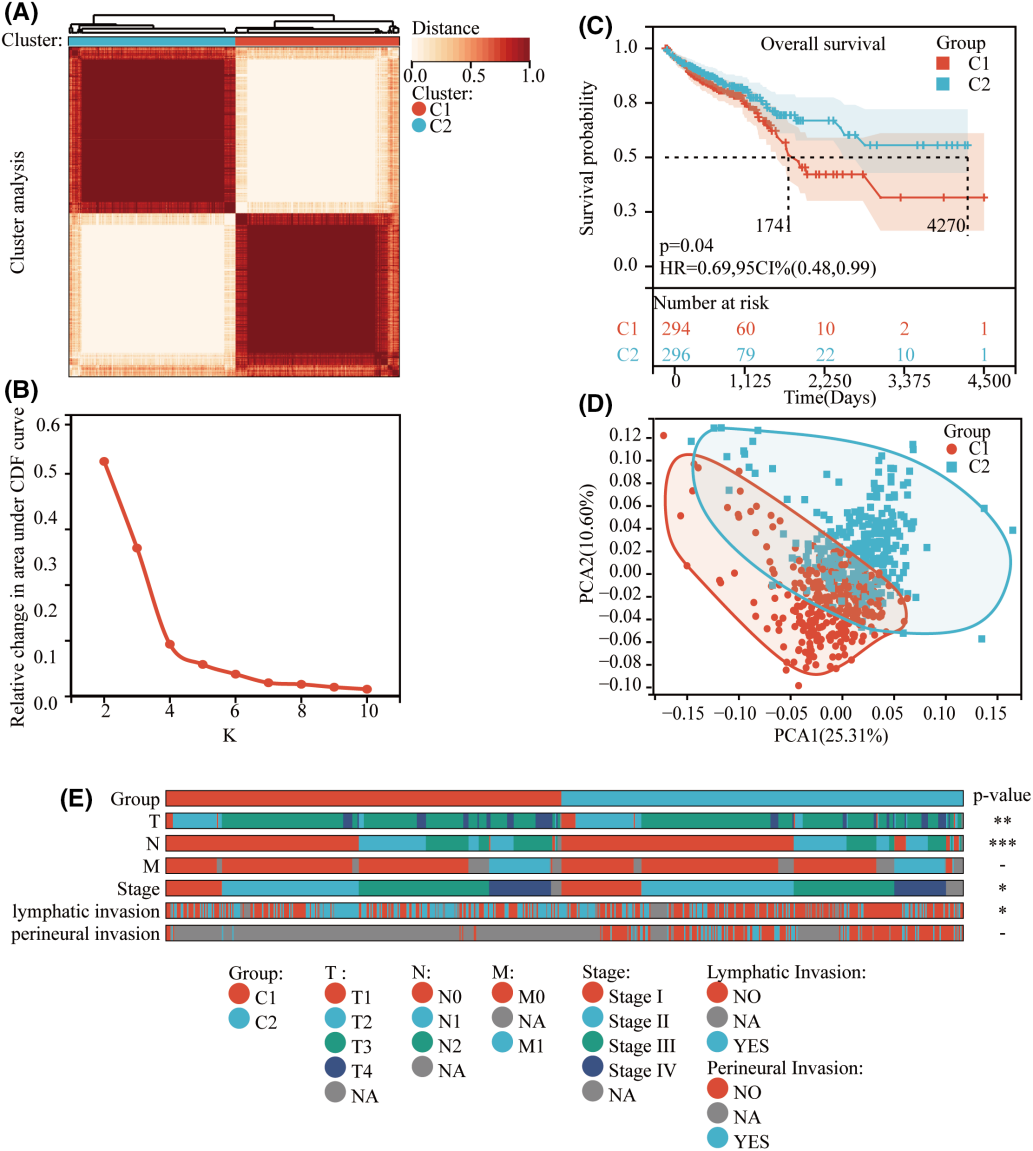
Classification based on the ACGs. A: 617 CRC patients were grouped into two groups according to the consensus clustering matrix (*k* = 2). B: CDF results from *k* = 2 to *k* = 10. C: Kaplan–Meier OS curves for the two groups. E: the clinicopathological features of the two groups

To investigate the role of actin cytoskeleton regulation in CRC, we first selected gene sets in KEGG pathway‐Regulation of Actin Cytoskeleton (hsa04810)^15^ and used expression data of gene sets mentioned above from TCGA‐CRC to cluster all samples into different k subtypes (*k* = 2–10) with Consensus Cluster Plus. Following the CDF findings (Figure 1B), we chose *k* = 2 as the best cluster number, implying that 617 CRC patients may be classified into two groups based on the ACGs (Figure 1A). According to the results of the survival analysis, C1 had a poor prognosis in terms of overall survival (OS) (Figure 1C). Furthermore, clinicopathological characteristics relating to tumor growth and invasion were collected for further investigation. Local invasion (*p* = 0.01), lymph node metastasis (*p* = 0.0013), clinical stage (*p* = 0.05), and local lymphatic invasion (*p* = 0.05) were all shown to be statistically significant differences between the two groups (Figure 1E).

### 3.2 Searching for key ACGs and constructing a prognostic model

**FIGURE 2:**
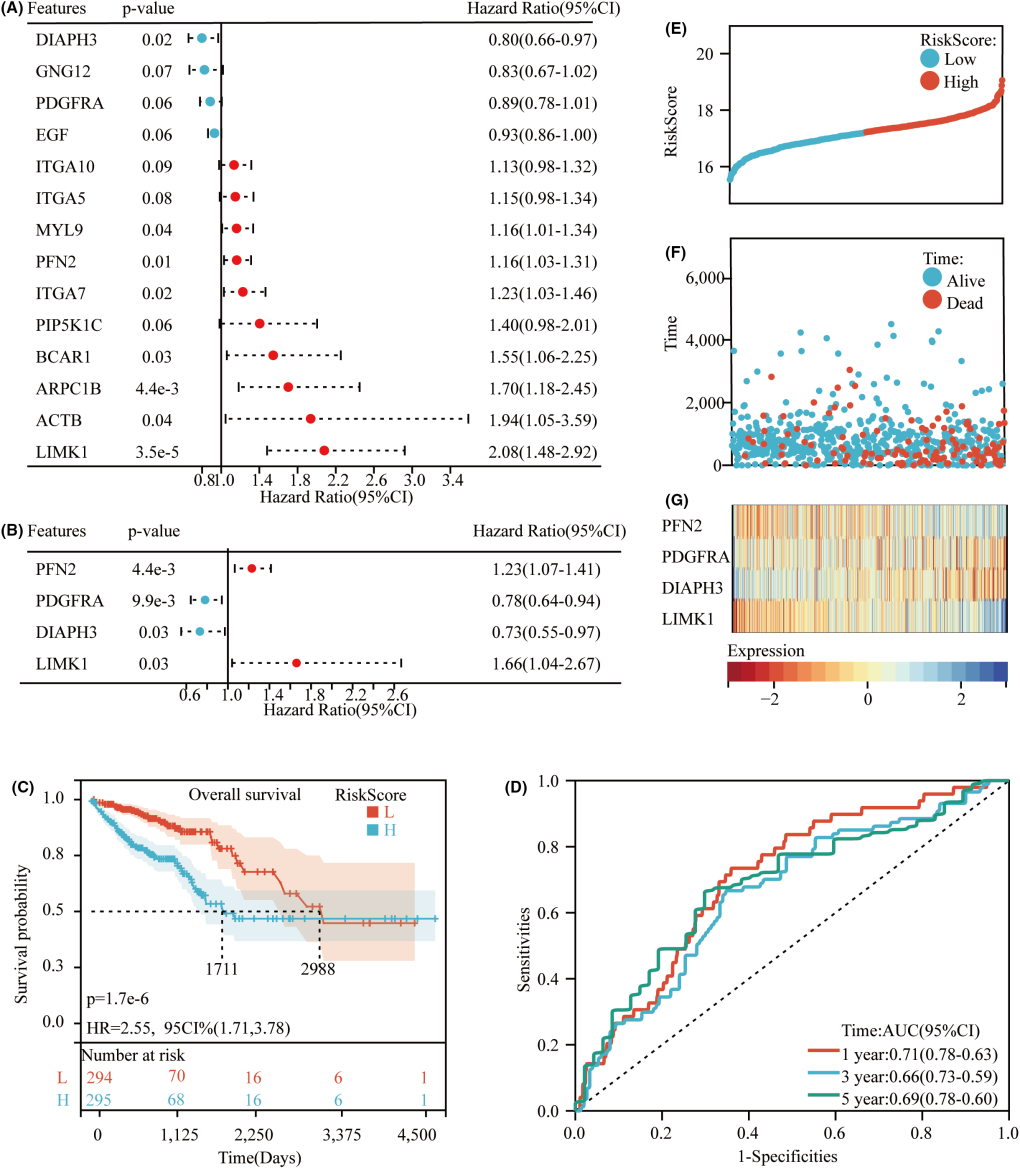
Construction of the risk signature in the TCGA cohort. A: Univariate Cox regression analysis of CRC for each ACG and 14 genes with *p* < 0.1. B: Multivariate Cox regression analysis of CRC with 14 Prognosis‐related genes and four Prognosis‐related ACGs were selected. C: Kaplan–Meier curves for the OS of patients in the high‐risk and low‐risk groups. D: ROC curves demonstrated the predictive efficiency of the risk score. E and F: Distribution and survival status f of each patient based on the risk score. G: Heatmap of the expression of the four Prognosis‐related ACGs in low‐risk and high‐risk groups

After finding that actin cytoskeleton regulation may play a critical role in prognosis and tumor progression in CRC, we next selected 617 CRC patients with complete survival data in TCGA for further analysis. First, we screened for prognosis‐related genes by univariate Cox. A total of 15 genes that met the *p* < 0.1 criteria were retained (Figure 2A). We selected the above genes for multivariate Cox regression and screened out four key ACGs: LIMK1, PFN2, PDGFRA, and DIAPH3 (Figure 2B). We then constructed a prognosis‐related model based on the above four genes and divided CRC patients into low‐risk group and high‐risk group according to the median risk score (Figure 2E–G). The results showed that high‐risk patients had a poor prognosis in terms of overall survival (OS) (*p* < 0.001) (Figure 2C). The predictive model constructed by the risk score was evaluated by ROC analysis. The area under the 1‐year, 3‐year, and 5‐year ROC curves (AUCs) is 0.71, 0.66, and 0.69, respectively (Figure 2D).

### 3.3 Expression of prognosis‐related ACGs in CRC

Since the prognostic model created above did not demonstrate a high degree of predictive accuracy, we switched our emphasis away from the high‐ and low‐risk grouping analysis based on this model and toward the model's ACGs. As a result, we began by examining the RNA expression levels of four genes in TCGA‐CRC using Gene Expression Profiling Interactive Analysis (GEPIA).^16^

**FIGURE 3:**
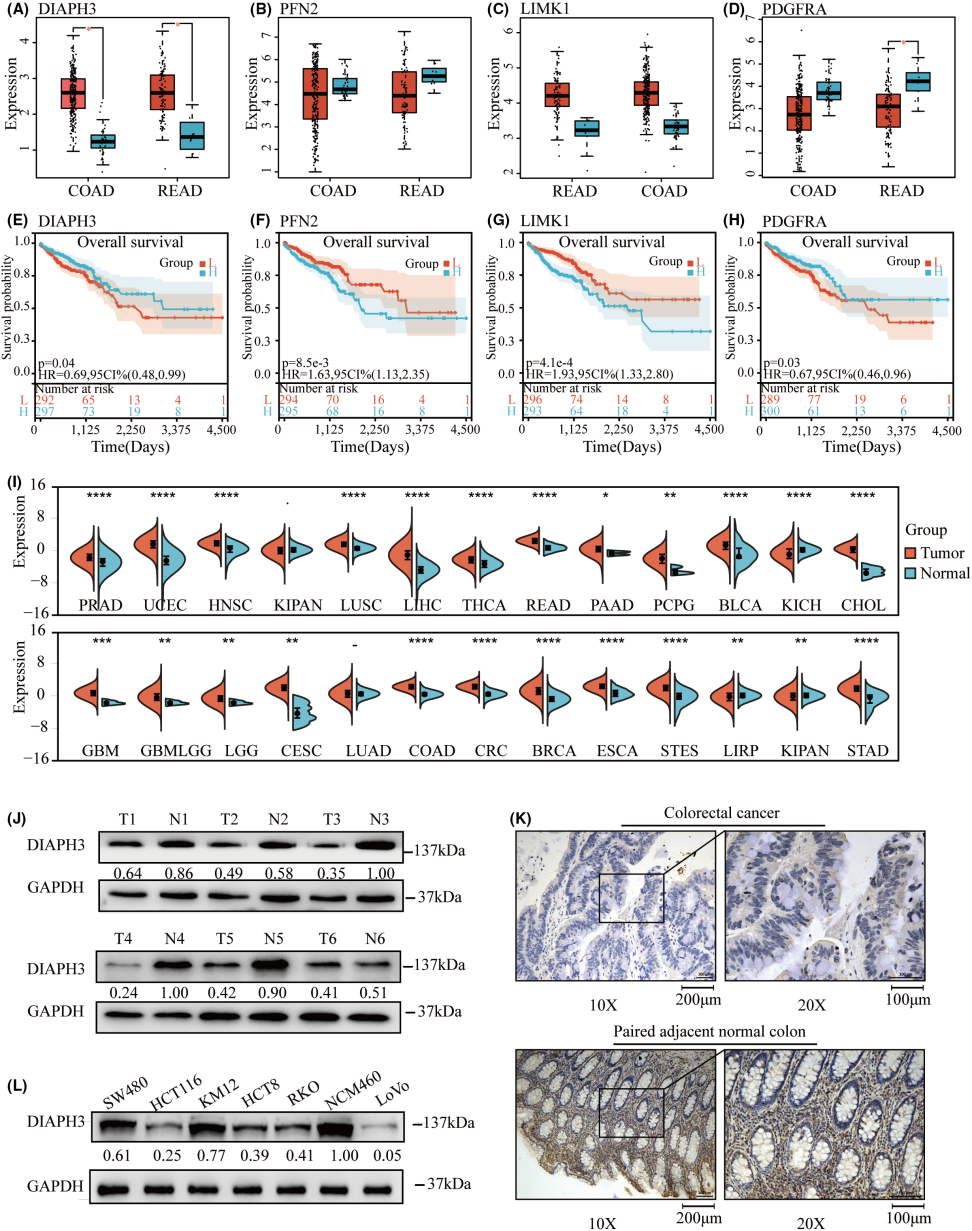
Expression of Prognosis‐related ACGs DIAPH3 in CRC. A–D: Expression of Prognosis‐related ACGs LIMK1, PFN2, PDGFRA, and DIAPH3 in TCGA‐COAD and TCGA‐READ. E–H: Overall survival (OS) of Prognosis‐related ACGs LIMK1, PFN2, PDGFRA, and DIAPH3 in TCGA‐COAD and TCGA‐READ. 3I: Expression of DIAPH3 of Pan‐cancer in TCGA. J and K: Western blot and Immunohistochemical staining for protein expression level of DIAPH3 in CRC and paired normal tissues tissue. L: Protein expression level of DIAPH3 in different CRC cells

We observed that DIAPH3 and LIMK1 were expressed in higher levels in CRC tumor tissue (Figure 3A,C), peri‐carcinomatous tissue expressed lower levels of PDGFRA (Figure 3D), and there was no significant difference in PFN2 expression between them (Figure 3B). Additionally, PFN2,^17^ LIMK1,^18^ and PDGFRA^19^ have been demonstrated to play critical roles in CRC. As a result, we investigated DIAPH3's function in CRC. To begin with, the overall survival curve shows that high levels of DIAPH3 expression in CRC are associated with a better prognosis, so DIAPH3 may act as a protective factor in CRC (Figure 3E). However, the level of expression in tumors is higher, so we investigated whether DIAPH3 is expressed in other cancers with similar conditions.

As a consequence, TCGA Pan‐Cancer was retrieved from the UCSC database (PANCAN, N = 10,535, G = 60,499) (https://xenabrowser.net) and we collected DIAPH3 gene expression data from a variety of samples, and log2 (*x* + 0.001) transformations were performed on each tumor sample's expression value. As shown in Figure 3I, DIAPH3 expression was found to be significantly higher in almost all tumors than in normal tissues adjacent to cancer, but the available literature revealed that its mode of action varied significantly between different cancers.^11^, ^12^, ^13^, ^20^ However, there was no literature to verify its expression and role in CRC. Therefore, we examined its expression using CRC tissue and cell lines. We observed that DIAPH3 expression is lower in cancer than in normal tissues adjacent to cancer (Figure 3J–K). Additionally, the degree of DIAPH3 protein expression varies significantly among cell lines (Figure 3L).

### 3.4 Knockdown of DIAPH3 promotes KM12 and SW480 cell proliferation and migration

**FIGURE 4:**
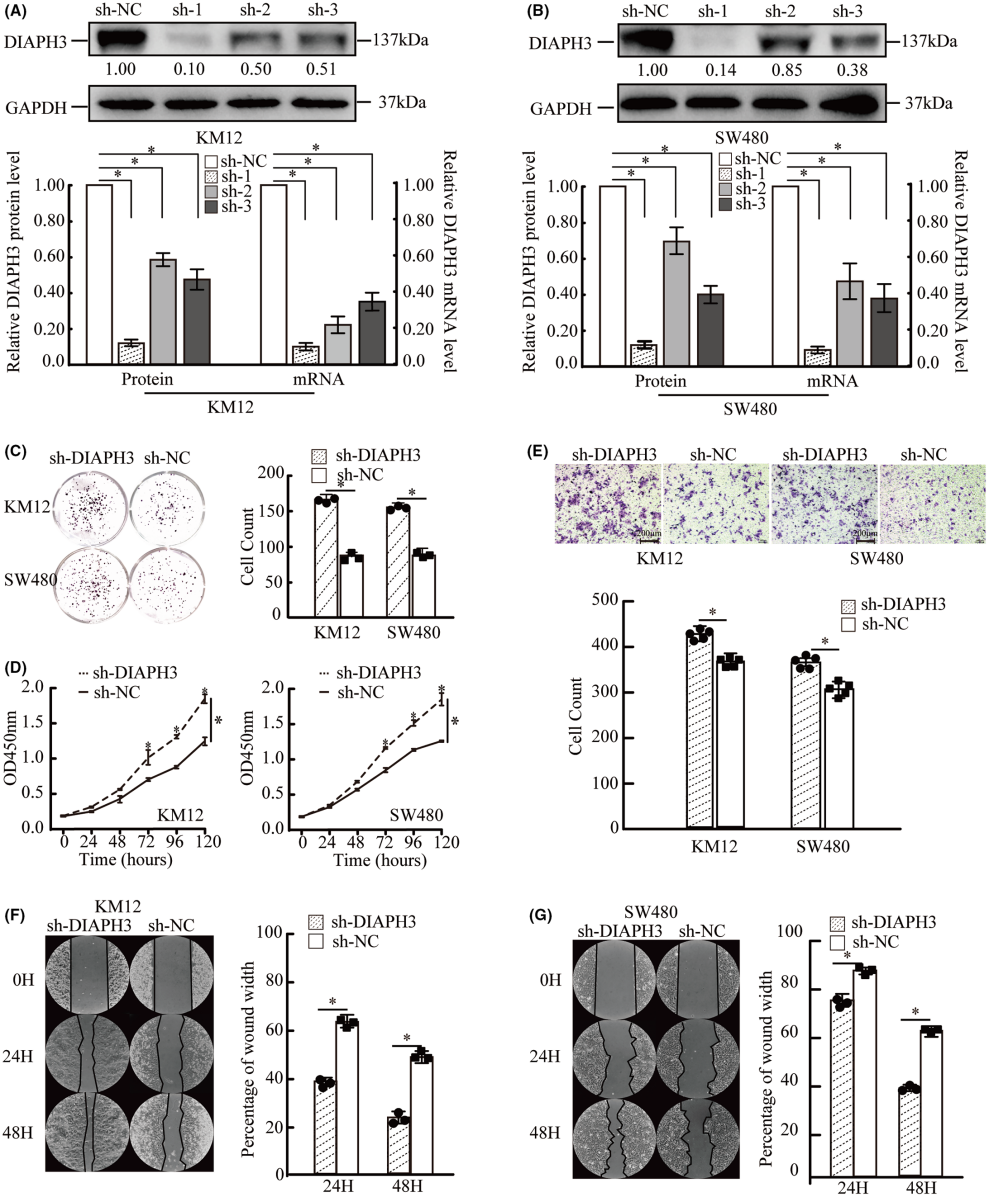
Knockdown of DIAPH3 promotes KM12 and SW480 cells proliferation and migration. A and B: Western blot was used to measure DIAPH3 expression after transfection with sh‐DIAPH3 ‐ and sh‐NC in sw480 and km12; ImageJ was used to analyze gray valve from blots above to quantify the protein expression, mRNA expression in paired group. GAPDH was used as internal control. (*t*‐test, *p < 0.05, *n* = 3). C and D: Plate clone formation assay and CCK8 assay were carried out to measure cell proliferation in each group. Data are expressed as the mean ± SD. (ANOVA *p < 0.05, *n* = 3. E: lower chamber KM12 and SW480 cells transfected with sh‐NC and sh‐DIAPH3, were captured with magnification ×100. Number of cells in five random fields were counted and averaged pre‐field. Data are expressed as the mean ± SD. (*t*‐test, *p < 0.05, *n* = 3). F and G: Wound healing assays indicated the effects of DIAPH3 suppression on the migration of CRC cell lines KM12 and SW480. Images were captured after 24 h and 48 h repaired and healing area was detected by ImageJ to analyze healing rates. Data are expressed as the mean ± SD, (*t*‐test, *p < 0.05, 3 repeated)

Next, we aim to determine if DIAPH3 is capable of controlling CRC development or migration in this situation. On the basis of the aforementioned findings, we hypothesized that DIAPH3 deficiency would enhance CRC migration and proliferation. As a result, we selected the SW480 and KM12 CRC cell lines for transfection to silence DIAPH3. Transfection efficiency was determined using western blot and RT‐qPCR assays. As seen in Figure 4A,B, fragment sh‐1 had the greatest effect on DIAPH3 protein and mRNA expression when compared to the empty vector sh‐NC. Following that, we chose sh‐1 (named sh‐DIAPH3) and sh‐NC to validate our prediction. Cell proliferation was measured by the cck8 assay. The results indicated that sh‐DIAPH3 SW480 and sh‐DIAPH3 KM12 cells proliferated significantly faster than sh‐NC cells (Figure 4C). Additionally, plate clone formation assays were used to confirm the findings. We noticed that silencing DIAPH3 was more efficient in terms of cloning rate in KM12 and SW480 cells (Figure 4D). Transwell and wound healing assays were used to determine the capacity of sh‐NC and sh‐DIAPH3 to migrate in SW480 and KM12 cell lines. Those findings suggest that sh‐DIAPH3 cells were more readily able to access the bottom chamber than sh‐NC cells in the SW480 and KM12 cell lines (Figure 4E). Additionally, wound healing assays indicated the same; the experimental group sh‐DIAPH3 had a greater potential for migration in SW480 and KM12 cells (Figure 4F).

### 3.5 DIAPH3 depletion may regulate the wound healing process and is involved in the EGFR pathway

**FIGURE 5:**
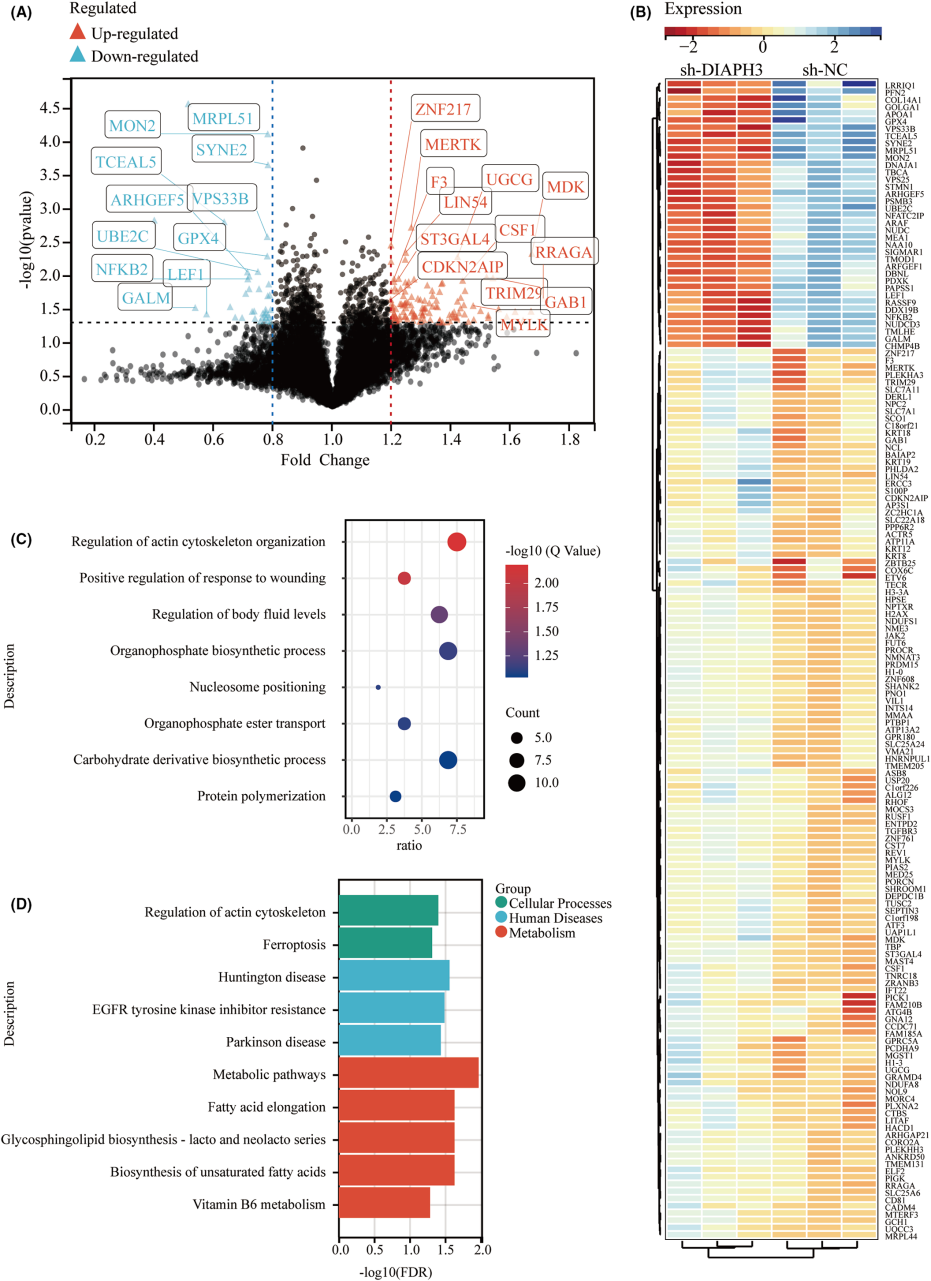
Differentially expressed proteins in TMT‐based quantitative proteome analysis on KM12 after DIAPH3 depletion. A: Volcano plot of differentially expressed proteins between sh‐DIAPH and sh‐NC in KM12. B: Heatmap for the differentially expressed proteins between sh‐DIAPH and sh‐NC in KM12. C: Bubble graph for GO enrichment of all differentially expressed proteins. D: Barplot graph for KEGG pathways of all differentially expressed proteins

After clarifying its function, we conducted quantitative proteome analysis on KM12 cells after DIAPH3 depletion to ascertain the effect of DIAPH3 interference. Hundred and sixty‐one significantly differently expressed proteins were discovered, as well as 123 upregulated and 38 downregulated differentially expressed proteins (Figure 5A,B). To gain a better understanding of the biological functions of the differentially expressed proteins, enrichment analyses were performed using GO and KEGG pathways. GO enrichment results revealed that its 161 differentially expressed proteins were enriched in Regulation of actin cytoskeleton organization (Figure 5C). The literature indicates that DIAPH3 may accelerate actin extension and stabilize tubulin, and since the domains of the two activities we volatilize overlap, it is not ruled out that the two actions are inhibited competitively.^21^ In tumor cells, decreased DIAPH3 expression restricts actin extension and results in aberrant microtubules, resulting in dramatic alterations in cell shape and increased cell motility. Additionally, we observed another GO enrichment result: wound healing (Figure 5C). Impairment of DIAPH3 function may lead in wound healing process, which is linked to major signaling pathways involved in metastatic tumor growth, including EGFR, TGF‐beta, and FGFR, of which EGFR is a critical component.^22^ Additionally, KEGG enrichment results suggest an EGFR‐related pathway‐EGFR tyrosine kinase inhibitor resistance (Figure 5D). As a result, we hypothesize that inhibiting DIAPH3 expression may enhance CRC proliferation and migration by EGFR activation.

### 3.6 Knockdown of DIAPH3 activates the EGFR, and its functions in cell proliferation and migration can be reversed by IMM‐01 and Gefitinib

**FIGURE 6:**
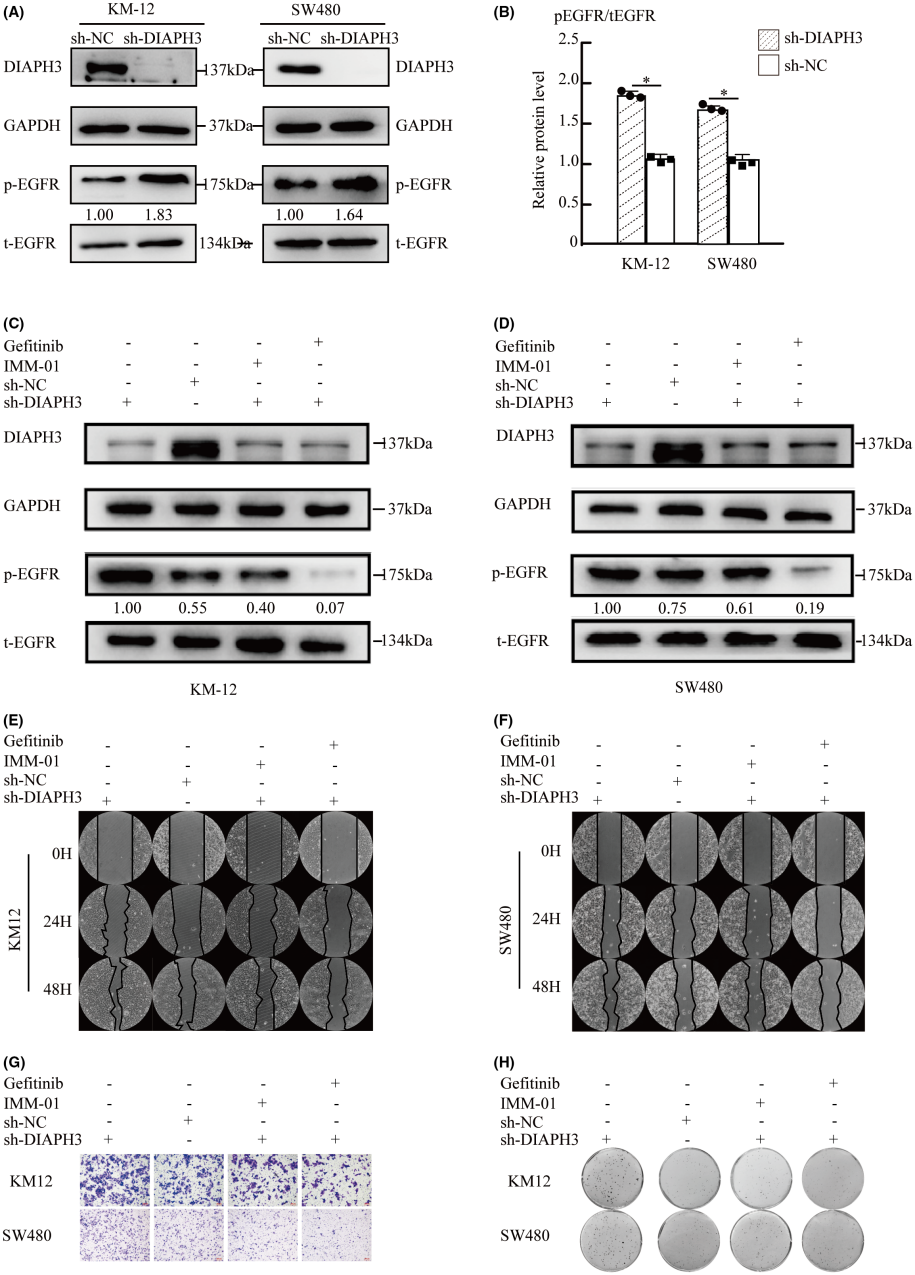
Activation of EGFR and Enhanced proliferation and migration ability caused by DIAPH3 depletion was revered by IMM‐01 and Gefitinib. A and B: sh‐NC and sh‐DIAPH3 in KM12 and SW480 cells were harvested, and expression of p‐EGFR, and EGFR was determined by western blot. GAPDH was used as internal control. *p < 0.05, *n* = 3; pEGFR/tEGFR and ImageJ was used for quantitative analysis of the blots. C and D sh‐DIAPH3 and sh‐NC treated 1 μM gefitinib or 1 μM IMM‐01 for 24 h were harvested, and expression of p‐EGFR, and EGFR was determined by western blot. GAPDH was defined as an internal control. E and F: Wound healing assays indicated the effects of migration ability of each group. Images were captured after 24 h and 48 h repaired and healing area was detected by ImageJ to analyze healing rates. Data are expressed as the mean ± SD, (*t*‐test, *p < 0.05, *n* = 3). G: lower chamber in each group was captured with magnification ×100. Number of cells in five random fields were counted and averaged pre‐field. Data are expressed as the mean ± SD. (*t*‐test, *p < 0.05, *n* = 3). H: Plate clone formation assay was carried out to measure cell proliferation in each group. Data are expressed as the mean ± SD. (*t*‐test, *p < 0.05, *n* = 3)

To verify our hypothesis, we extracted protein from each group and detected total EGFR and phosphorylated EGFR using western blot. GAPDH was used as an internal control. ImageJ was used for quantitative analysis of the blots. As shown in Figure 6A, inhibition of DIAPH3 could stimulate EGFR. Moreover, FH1‐FH2 (code domain of DIAPH3) targeted activator IMM‐01 was used to verify the conclusion. Western blots indicated that IMM‐01 could reverse the activity of EGFR. Then we used EGF to stimulate and we found that after knockdown of DIAPH3, the EGFR pathway can be continuously active (Figure 6C,D).

Next, we repeated the relevant experiments to verify the effects on cell proliferation and migration under IMM‐01. First, 1 μM IMM‐01 was added to cultures, and then Transwell, wound healing, and clone formation assays were performed to detect changes in cell proliferation and migration. As shown in Figure 6E–H, IMM‐01 can reverse in DIAPH3 exhausted colorectal cell lines, which leads to the increased ability of the cell proliferation, migration, which was consistent with the treatment on EGFR pathway inhibitor gefitinib.

### 3.7 DIAPH3 may involve in maintaining EGFR degradation, and its downregulation leads to intracellular accumulation of EGFR

**FIGURE 7:**
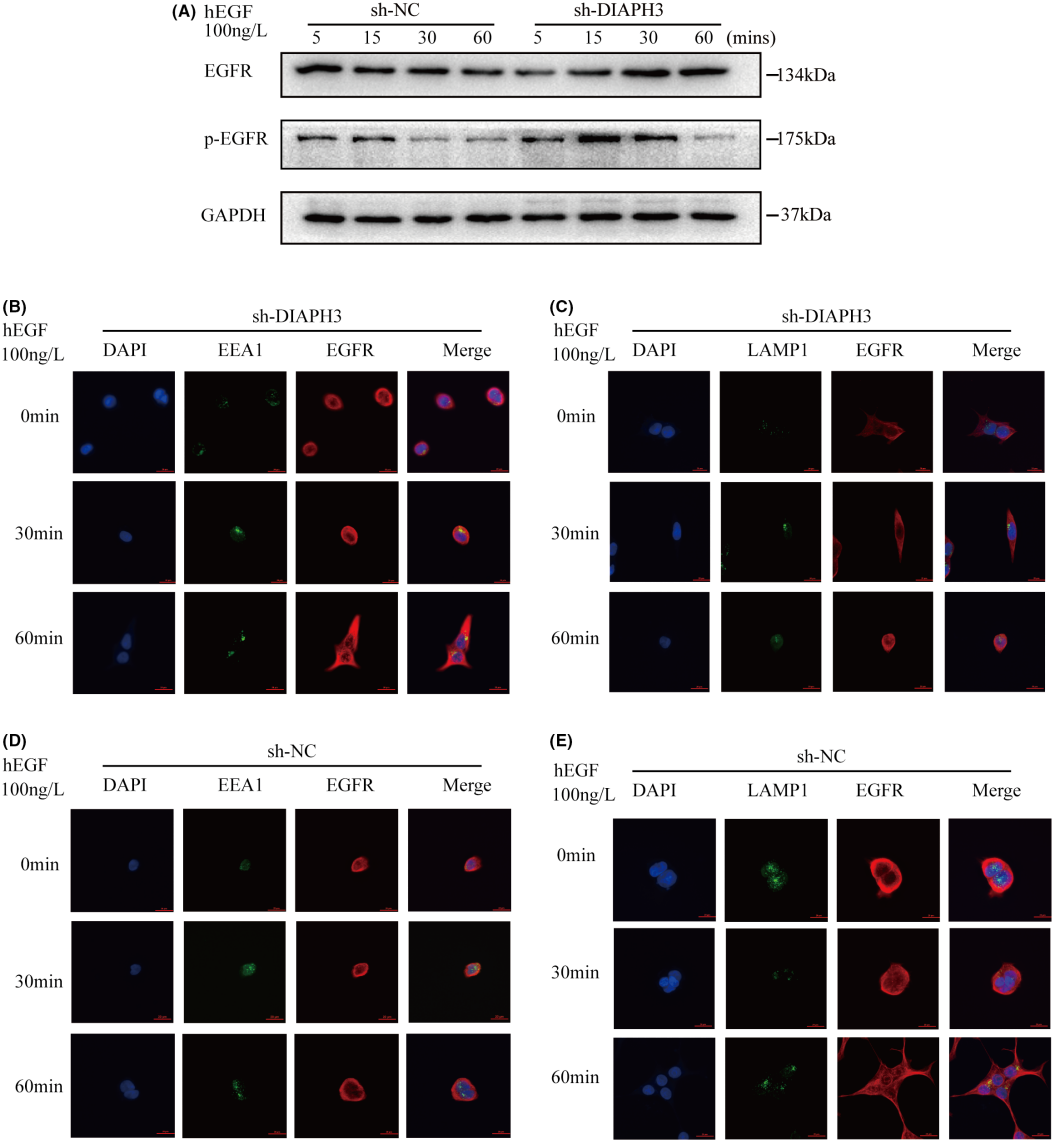
Western blot and immunofluorescence results of EGFR activation under the stimulating with hEGF in sh‐DIAPH3 and sh‐NC cell. A: sh‐NC and sh‐DIAPH3 in KM12 cells were harvested while treating with 100 ng/ml hEGF for 5, 15, 30, 60 min and expression of p‐EGFR, and EGFR was determined by western blot. GAPDH was used as internal control. B and D Immunofluorescence stain of EGFR (Red) and EEA1 (Green) of sh‐NC and sh‐DIAPH3 in KM12 while treating with 100 ng/ml hEGF for 0, 30, 60 min. C and E Immunofluorescence stain of EGFR (Red) and LAMP1 (Green) of sh‐NC and sh‐DIAPH3 in KM12 while treating with 100 ng/ml hEGF for 0, 30, 60 min

After demonstrating that DIAPH3 can influence EGFR activation, we utilized EGF to stimulate and discovered that after DIAPH3 depletion, the EGFR pathway may remain active indefinitely (Figure 7A). In this respect, we used human epidermal growth factor (hEGF) to stimulate sh‐DIAPH3 and sh‐NC cells and performed immunofluorescence to detect EGFR internalization. We discovered that when DIAPH3 expression is inhibited, hEGF stimulation increases EGFR binding to early endosomes (Figure 7B,D) but decreases binding to lysosomes (Figure 7C,E). Hence, DIAPH3 may be involved in regulating the endocytotic degradation of EGFR.

## 4 DISCUSSION

In our study, we used gene sets in KEGG pathway‐Regulation of Actin Cytoskeleton to classify CRC patients into two groups. Significant disparities in clinicopathological characteristics such as T‐class tumor stage, lymph node metastasis, clinical stage, and local lymphatic invasion were seen across the two groups.

All sorts of cell motility and extracellular structural alterations are influenced by actin and cytoskeletal activity. Previous research has shown that multi‐factored, complex molecular biology changes caused by actin cytoskeleton are involved in cancer invasion and metastasis, such as cell adhesion,^23^, ^24^ epithelial‐mesenchymal transition,^25^ mesenchymal amoeboid transition,^26^ and the degradation of extracellular matrix.^27^ However, it is unknown how to accomplish these adjustments in CRC by modifying the actin cytoskeleton in such a way that cells may modify their motor patterns in response to these triggers. This study revealed four predictive biomarkers: LIMK1, PFN2, PDGFRA, and DIAPH3. PFN2^17^ and LIMK1^18^ are implicated in EMT, whereas PDGFRA is a therapeutic target in young CRC patients,^19^ and its mutations may contribute to the development of CRC.^28^

Furthermore, we concentrated on DIAPH3 and concluded that DIAPH3 acts as a cancer suppressor in CRC. The depletion of DIAPH3 is also related to tumor progression and poor prognosis. DIAPH3 is oriented in the lamella of migrating epithelial cells where it participates in maintaining cortical actin pool stability. Its inhibition slows the focal adhesion decomposition rate and alters the dynamics and organization of F‐actin, which are indispensable for efficient epithelial cell migration.^29^ Cell migration is the cornerstone of tumor invasion and metastasis. The process involves morphological polarization, cell front membrane extension, adhesion of extracellular matrix, and the forward contraction of the cell membrane and release of the posterior part engaged in cell adhesion.^30^ Each step of the migration is mediated by a different dynamic actin filament F‐actin. Thus, migration speed was reduced because of DIAPH3 inhibition.^29^ This is exactly the opposite of the function of DIAPH3 in tumor cells. Competitive inhibition may exist in those two functions, or they may not be performed simultaneously. There is a possibility that in normal epithelial cells, the FH2 domain in DIAPH3 employs actin polymerization to maintain the rate of focal adhesion for stabilizing migration at the leading edge of the cell. However, in tumor cells, DIAPH3 depletion leads to abnormal activity of the microtubule, which causes violent changes in morphology, enhances cell mobility, and promotes cell migration and those functions may not be mutually exclusive. Research indicated that extravasated cancer cells could mediate the Rif/DIAPH3 pathway to initiate filopodium‐like protrusions (FLPs). Additionally, FLPs interacts with EMC components such as integrin‐β1 to potentiate adhesion plaque assembly, which facilitates metastatic cell recolonization in the liver.^31^ This indicates that the function of DIAPH3 may be regulated by several upstream factors. In tumor cell migration, DIAPH3 was shown to be involved in several important processes, including the formation of extracellular vesicle,^32^ amoeboid mode invasion, and participation in tumor microenvironment (TME) with carcinoma‐associated fibroblasts.^33^

In this paper, we demonstrate the possible mechanisms of DIAPH3 in activating EGFR to promote proliferation and metastasis in CRC. However, we did not reveal the direct connection between them. Recently, proteomics has also shown us the novel phenotype of DIAPH3 in tumor regulation, including acting as a role for ubiquitin for regulating proteasome activity. One possibility is that DIAPH3 depletion destroys endocytosis and may induce EGFR accumulation in endosomes. Thus, the lysosome transfer and membrane circulation of the EGFR are relieved so that EGFR can maintain persistent activation, triggering the downstream factors.

Moreover, IMM‐01 can target the diaphanous inhibitory domain (DID) and restrain the self‐restraining ring formation in DIAPH3 to maintain its functions.^34^ We found that IMM‐01 can reverse the promotion of migration ability in DIAPH3‐deleped CRC cells. Further, it inhibits the activity of EGFR. Thus, our results suggest that IMM‐01 can be useful as an alternative targeted therapy for patients with EGFR‐targeted drug resistance. Therefore, the mechanism of DIAPH3 is worth exploring in CRC. We also hope to be able to transform our harvest from basic research to benefit clinical patients.

## AUTHOR CONTRIBUTIONS

ZZW, YJL, and HRL conceived and designed the study; ZZW, HRL, WJL, and WC performed the experiments; HRL and ZZW performed the data analyses and wrote the manuscript; ZHD and YJY helped perform the analysis with constructive discussions. YJY, WC, and YJY reviewed and edited the manuscript. All authors read and approved the manuscript.

## CONFLICT OF INTEREST

The authors declare that they have no conflict of interest.

## ETHICAL APPROVAL STATEMENT

This research was approved by Research ethics committees of ZhuJiang hospital, Southern medical university, each patient in this research signed written informed consent, authorized their specimens for scientific research. Their privacy will be fully maintained.

## Supporting information


Data S1

Table S1

Table S2

## Data Availability

The Cancer Genome Atlas was used in this investigation. Additional data linked with this paper have been uploaded as “Supplementary Table S1”, “Supplementary Table S2”, and “Supplementary Data S1”. Other datasets generated and analyzed during the current study are available from the corresponding author upon reasonable request.
