# Supplementary material for: DIAPH3 is a prognostic biomarker and inhibit colorectal cancer progression through maintaining EGFR degradation
Source: Cancer Med. 2022 May 11;11(23):4688–702. doi: 10.1002/cam4.4793 (PMC9741984; doi:10.1002/cam4.4793)
Supplement: Supplementary file 1 — Data S1 [file CAM4-11-4688-s002.docx]

Supplementary document 3

| RNA interference | Sequence |
| --- | --- |
| DIAPH3-sh1 | CCGGCGTGTCAGAATAGCTAAAGAACTCGAGTTCTTTAGCTATTCTGACACGTTTTTTG |
| DIAPH3-sh2 | CCGGCCAGATTTGTTATCAGTGCAACTCGAGTTGCACTGATAACAAATCTGGTTTTTTG |
| DIAPH3-sh3 | CCGGGCATGACAAGTTTGTGACAAACTCGAGTTTGTCACAAACTTGTCATGCTTTTTTG |

Sequence of RNA interference

| qPCR | Sequence |
| --- | --- |
| DIAPH3-Forward | 5′-GTTCGGCAGAGTCTCAGTCCAATG- 3′; |
| DIAPH3-Reverse  GAPDH-Forward | 5′-CTTGGCGACTGGAGTCCTTGTTG-3′;  5′-CATCACCATCTTCCAGGAGCG -3′; |
| GAPDH-Reverse | 5′-TGACCTTGCCCACAGCCTTG -3′. |

qPCR Primer

The primary antibodies included: rabbit anti-human DIAPH3 mAb (working dilutions: 1:800; Code:14342-1-AP, Proteintech, China), mouse anti-human EGFR mAb (working dilutions:1:2000; Code:18986-1-AP, Proteintech, China), mouse anti-human GAPDH mAb (working dilutions: 1:5000; Code: 60004-1-Ig, Proteintech, China), rabbit anti-human p-EGFR mAb (working dilutions: 1:500; Code: 4407S, CST, USA), rabbit anti-human EEA1 mAb (working dilutions: 1:100; Code: 2411S, CST, USA), rabbit anti-human LAMP1 mAb (working dilutions: 1:100; Code: 9091T, CST, USA),
